# The Teamwork Assessment Scale: A Novel Instrument to Assess Quality of Undergraduate Medical Students' Teamwork Using the Example of Simulation-based Ward-Rounds

**DOI:** 10.3205/zma000961

**Published:** 2015-05-13

**Authors:** Jan Kiesewetter, Martin R. Fischer

**Affiliations:** 1 Klinikum der LMU München, Institut für Didaktik und Ausbildungsforschung in der Medizin, München, Germany; 2 Ludwig-Maximilians-Universtiy Munich, Munich Center of the Learning Sciences (MCLS), Munich, Germany

**Keywords:** simulation-based training, team training, team performance measurement, medical education, teaching and learning

## Abstract

**Background:** Simulation-based teamwork trainings are considered a powerful training method to advance teamwork, which becomes more relevant in medical education. The measurement of teamwork is of high importance and several instruments have been developed for various medical domains to meet this need. To our knowledge, no theoretically-based and easy-to-use measurement instrument has been published nor developed specifically for simulation-based teamwork trainings of medical students. Internist ward-rounds function as an important example of teamwork in medicine.

**Purposes: **The purpose of this study was to provide a validated, theoretically-based instrument that is easy-to-use. Furthermore, this study aimed to identify if and when rater scores relate to performance.

**Methods: **Based on a theoretical framework for teamwork behaviour, items regarding four teamwork components (*Team Coordination, Team Cooperation, Information Exchange, Team Adjustment Behaviours*) were developed. In study one, three ward-round scenarios, simulated by 69 students, were videotaped and rated independently by four trained raters. The instrument was tested for the embedded psychometric properties and factorial structure. In study two, the instrument was tested for construct validity with an external criterion with a second set of 100 students and four raters.

**Results:** In study one, the factorial structure matched the theoretical components but was unable to separate Information Exchange and Team Cooperation. The preliminary version showed adequate psychometric properties (Cronbach’s α=.75). In study two, the instrument showed physician rater scores were more reliable in measurement than those of student raters. Furthermore, a close correlation between the scale and clinical performance as an external criteria was shown (r=.64) and the sufficient psychometric properties were replicated (Cronbach’s α=.78).

**Conclusions: **The validation allows for use of the simulated teamwork assessment scale in undergraduate medical ward-round trainings to reliably measure teamwork by physicians. Further studies are needed to verify the applicability of the instrument.

## Zusammenfassung

**Hintergrund: **Simulationsbasierte Teamarbeitstrainings werden als eine wirkungsvolle Methode betrachtet um Teamarbeit, zu verbessern. Teamarbeit wird in der medizinischen Ausbildung immer relevanter. Auch die Messung von Teamarbeit ist daher sehr wichtig und verschiedene Instrumente wurden für verschiedene medizinische Fachdisziplinen entwickelt, um diesen Zweck zu erfüllen. Unseres Wissens nach ist bisher kein theoriebasiertes und einfach anzuwendendes Messinstrument spezifisch für simulationsbasierte Teamarbeitstrainings von Medizinstudierenden publiziert oder entwickelt worden. Internistische Visiten fungieren als ein wichtiges Beispiel von Teamarbeit in der Medizin.

**Ziel: **Das Ziel der Studie war ein validiertes, theoriebasiertes Instrument zu entwickeln, welches einfach anzuwenden ist. Weiterhin sollte in der Studie untersucht werden, ob und wann die Bewertungen von Ratern mit klinischer Performanz in Zusammenhang stehen.

**Methoden:** Basierend auf einem theoretischen Rahmenmodell für Teamarbeitsverhalten wurden Items zu vier Teamarbeitskomponenten entwickelt *(Teamkoordination, Teamkooperation, Informationsaustausch, Verhaltensweisen zur Teamanpassung*). In Studie eins wurden drei Visitenszenarien, simuliert von 69 Studierenden, gefilmt und unabhängig von vier trainierten Ratern bewertet. Das Instrument wurde auf seine psychometrischen Eigenschaften und die faktorielle Struktur hin untersucht. In Studie zwei wurde die Konstruktvalidität des Instruments anhand eines externen Kriteriums mit einem zweiten Set von 100 Studierenden und vier Ratern überprüft.

**Ergebnisse:** In Studie eins passte die faktorielle Struktur zu den theoretischen Komponenten, jedoch konnte Informationsaustausch und Teamkooperation nicht separiert werden. Die vorläufige Version zeigte ausreichende psychometrische Eigenschaften (Cronbach’s α=,75). In Studie zwei waren die ärztlichen Bewertungen reliabler als die studentischen Bewertungen. Weiterhin konnte eine enger Zusammenhang zwischen der Skala und klinischer Performanz als einem externen Kriterium (r=,64) gezeigt werden und die ausreichend hohen psychometrischen Eigenschaften konnten repliziert werden (Cronbach’s α=,78).

**Schlussfolgerungen:** Die Validierung gestattet den Einsatz der Teamarbeitsskala in Visitentrainings von Studierenden, um Teamarbeit reliabel von Ärztinnen und Ärzten einschätzen zu lassen. Weitere Studien sind notwendig, um die Anwendbarkeit des Instruments zu verifizieren.

## Introduction

Insufficient communication and teamwork are among the major causes for sentinel events in healthcare [1], [2]. Furthermore, errors in teamwork have an enormous influence on patient safety [3], [4], [5], [6]. As a consequence of the aftermath of healthcare errors [7], the advancement of patient safety through improvement of teamwork is desirable and under intense discussion. To this end, simulation-based training is considered a powerful training method for team performance [8], [9]. 

In this article, we will introduce the *Teamwork Assessment Scale* (TAS), a teamwork measurement instrument built on a concise theoretical framework to reliably measure undergraduate training. In the following paragraphs, we

1. introduce the teamwork model on which we built our scale and
2. discuss existing teamwork measurement instruments.

We conclude the introduction by formulating how the theoretical model can help to develop a measurement instrument that goes beyond the applicability of the existing measurement instruments. 

In the existing literature, several teamwork models [10] have been established to capture teamwork behaviour. Rousseau, Aubé, and Savoie [10] developed a teamwork framework, which is derived from an analysis of twenty-nine teamwork models and arranged the included teamwork behaviours hierarchically. The integrated framework for teamwork behaviour is considered to be a comprehensive starting point for the development of a teamwork measurement instrument. Within the framework itself, teamwork behaviours are subdivided into the categories

1. *Regulation of team performance* and
2. *Management of team maintenance* (i.e. interpersonal difficulties among team members).

Within the *Regulation of team performance* category, the main behavioral determinants for a team’s success include

1. Preparation of Work Accomplishment,
2. Work Assessment Behaviours,
3. Team Adjustment Behaviours, and
4. Task-related Collaborative Behaviours

as the main behavioural determinants for a team’s success. Theoretically, we aim to build our measurement scale on this model so that the instrument is a strong predictor for a team’s performance. 

We will now discuss the current measurement instruments for teamwork. As has been recently described [11], critics of existing outcome measurement instruments in simulation-based training state that the behavioural spectrum of measurement is too narrow to fully capture today’s complex medical practice. An open empirical question is the relationship between measured teamwork and the quality of healthcare provided in a simulation-based environment (i.e. clinical performance). Subsequently, measurement development is a “high-priority issue” [11] in simulation-based medical education for two reasons:

1. to provide accurate feedback to learners and
2. to advance medical teamwork research with valid research results [11].

At present, however, the instruments presumably differ largely to the extent that they require prior knowledge from the rater. Furthermore, at times, the existing instruments lack careful theoretical foundation and construct validation. Construct validation indicates whether a scale measures and correlates with the theorized construct. The instruments for assessing the quality of teamwork are often developed for a specific medical subdiscipline (e.g. surgery [12], anesthesia [13], or emergency medicine [14], [15], [16], [17], [18]). Within these subdisciplines, the instruments work well. However, the instruments are not intended for use across medical disciplines and not designed to support learning of undergraduate students in a simulated team environment. 

Ward-rounds in diverse subdisciplines, however, are a context of everyday medical care in which medical professionals (i.e. doctors and nurses) have to work together efficiently as a team. For clinical care, there does exist an overall measurement checklist for ward rounds [18]. However, this checklist was especially designed for postgraduate training and no instrument is available for the undergraduate level. As ward-rounding is a complex key example of a 1^st^ day teamwork competency in clinical medicine, it seems important to already teach this skill to undergraduate students and to verify the quality of these trainings, which are often delivered in simulation-based environments. 

In the following paragraph, we will present how the integrated framework of teamwork behaviours by Rousseau et al. [10] can help to construct a teamwork scale that goes beyond one medical discipline and can be used for undergraduate training as well. As an example, we use the clinically relevant subject of ward-rounds in undergraduate medical education.

The component *Regulation of Team Performance* is an important aspect before and during most ward-rounds. Before the ward-round begins, the *Work Assessment Behaviours* could include the preparation of patient charts by the nurse. *Team Adjustment Behaviours* may be crucial if - unexpectedly - the patient shows new symptoms during a ward-round. *Task-related collaborative behaviours* between the team members would then facilitate the sharing of the patient-relevant information. Indeed, ward-rounds are complex tasks [19] but the roles, objectives, and activities of each member and the typical sequence of activities are predefined. Together, these roles, objectives, and activities can be identified as a collaboration script (cf. [20]) and fulfilment of such a ward-round script can be used as an external index for the clinical performance of the team. For the purposes of the measurement instrument, this index can help to verify the validity of the scale - the Teamwork Assessment Scale (TAS) - within ward-rounds.

The goal of this study was to develop an instrument based on a theoretical foundation that can be used in healthcare to:

1. measure teamwork components reliably in simulation-based scenarios designed for undergraduate students’ learning,
2. compare different team performances, and
3. evaluate the effect of teamwork trainings.

To achieve these goals, two studies were conducted to validate TAS: The first study aimed to evaluate psychometric properties of the items, and the second study aimed to determine the construct validity (using clinical performance as an external validation criterion) and evaluate which prerequisites reflect actual teamwork.

## Methods

### Development of the initial item set

Based on the hierarchical model, a simulated teamwork measurement instrument was developed: TAS. Different behaviour-based scales to observe teams in healthcare were reviewed [13], [15], [16], [17], [21]. The instruments were chosen as a sound starting point for the behaviours regarding *Task-related collaborative behaviours* (Coordination, Collaboration and Information exchange) and *Team Adjustment Behaviours*. Team Coordination relates to the integration of the members‘ roles and activities (cf. [22]), and is central to ward-rounds since the team’s members are assigned relatively spontaneously. Team Coordination relates to the integration of the members’ roles and activities (cf. [22]). Thus, the Team Cooperation demonstrated in simulated scenarios can aid in developing behaviors required for the completion of interdependent jobs in actual ward-round scenarios (cf. [23]). For instance, Information Exchange, which we define as the assigning and directing of tasks (cf. [24]), is important as teams in (actual and) simulated scenarios often begin with an imbalance of information that has to be resolved during the scenario. Team Adjustment Behaviours are defined as the teams’ activities to face unforeseeable performance demands (cf. [10]). The theoretical foundation and definition for the development of the measurement instrument is summarized in Table 1 (Tab. 1).

Initially, six to seven items per subscale (26 items overall) were developed in German and formulated to adapt to the simulation-based learning context of students . A five point Likert scale was applied*. 

#### Item Revision

All items were reviewed thrice by members of an interdisciplinary research team that consisted of medical professionals (n=3), educators (n=2), and psychologists (n=2). Afterwards, the items were included into the revised version. These professions were chosen to guarantee authenticity (medical professionals), applicability in training contexts (educators), and psychometric interpretability (psychologists). Each review included a portion for the research team to provide feedback independently on the current version items; the comments for which were summarized by one of the authors (J.K.) and subsequently discussed in a group discussion regarding their overall applicability.

The first version of the scale was piloted in the simulation clinic of the university hospital with three real-time, healthcare teamwork scenarios, which consisted of four doctors and one nurse. Five expert observer (whom were not included in the initial development) rated the healthcare teamwork scenarios. A discussion followed determining whether the individual items were:

1. representative, in that no obvious part of teamwork behaviours important for learning in simulated settings was completely left out,
2. observable, in that one with knowledge of teamwork could distinguish between teams who showed the behaviour and teams who did not, and
3. comprehensible, in that one with knowledge of teamwork literature could understand what the items mean and distinguish between the scales included in the instrument.

The group discussed the rating scale until every member of the research team agreed that the, partly reformulated, 17 items were representative, observable, and comprehensible.

#### Ethical Approval

Both studies reported here were approved by the faculties’ ethical committee (EK7 110-11).

## Evaluation Study 1: Scale Properties

The objective of Study 1 was to evaluate the instrument’s psychometric properties and verify the factorial structure regarding the authors’ conceptualization of teamwork according to the model by Rousséau et al. [10]. 

### Participants in the study

Twenty-one teams of three to four students (69 students in total: mean age=22.6; f=64%, m=36%) participated in this study in exchange for course credit. The students had an overall mean of 8.2 practical weeks in wards but had not yet worked together in a clinical setting. The overall sample was split into groups of twelve (i.e. three teams) with whom three teamwork scenarios were simulated. The non-simulating participants observed through a semi-permeable mirror while another team participated in the simulated teamwork scenario.

#### Simulated teamwork scenarios

The three scenarios were ward-rounds that differed in their complexity with regard to the quantity of information that had to be handled before and within the simulation. In scenario one, the patient is about to be discharged when the ECG on the ward round unexpectedly showed T-wave negativations. The team has to identify the ECG for this abnormality and discuss the new finding and its consequences with the patient. In scenario two, the patient was supposed to be fasting but had breakfast and, consequently, an ultrasound of the gastrointestinal tract scheduled for that morning has to be cancelled. This new information, potentially leading to conflict has to be identified and a solution needs to be found. In scenario three, a non-compliant patient refuses to take his medication. The team has to deal with the daily patient information and give the patient further diabetes education.

All simulated teamwork scenarios were conducted in real-time in the simulation clinic of the university hospital, where each scenario took between five and seven minutes to be carried-out. Each scenario contained three different roles: nurse, resident, and chief of medicine. The other roles (nurses and residents) were carried-out by medical students, and the role of the chief of medicine was always carried-out by a prepared and trained doctor in order to assure a high-level of standardization and conceptual proximity to actual teamwork.

#### Procedure

In an introductory session, all participants received information regarding the course and their roles, and were instructed not to talk about their roles during the days in-between. One week later, the teams carried-out their simulation and received a debriefing after their scenario. The order of the scenarios was balanced and all simulated scenarios were videotaped.

#### Data sources and instruments

After all simulations were finished, the videos were rated independently by four trained observer using the revised version of the TAS (17 items). The order in which the videos were presented was randomised to minimize any likelihood of order effects. All items were applied to a 5-point Likert scale (1=Completely Disagree, 5=Completely Agree). The ratings of three items were reversed (e.g. “In situations where team members needed help, it was not given to them.”) as low ratings indicated good teamwork behaviour.

#### Statistical Analysis

The rating of all items were z-standardized. The scale’s internal consistency was evaluated using Cronbach’s a. An explanatory factor analysis (Rotated Maximum Likelihood with Kaiser Normalisation) was used to test for dimensionality. 

## Results of Study 1

The overall mean for study one is M=3.73 with a standard deviation of SD=.70 before the factors were extracted (see Table 2 (Tab. 2)). The explanatory factor analysis showed four major factors for examination (Eigen-value>1). To refine the items, the following strategy was applied: First, only those items that loaded at least .40 on one of the primary factors were further included; second, items that cross-loaded at ≥.50 were eliminated (see Table 3 (Tab. 3)). Finally, redundant items (Cronbach’s α over .96) were eliminated. 

This strategy resulted in 14 items and revealed three factors expected from the theoretical foundation of the instrument: *Team Adjustment Behaviours* (TAB) as both intended and defined in Rousseau [10]; *Team Coordination* (TC) per the coordination and variation of the roles in the simulated scenario; and *Cooperation and Information Exchange* (CIE), an aggregated and theoretically plausible factor of the behaviours regarding *Team Cooperation* and *Information Exchange*.

Overall, both the resulting 14-item scale and the individual subscales showed good internal consistency (scale: Cronbach’s α=.754; *TC*: Cronbach’s α=.813; *CIE*: Cronbach’s α=.763; and *TAB*: Cronbach’s α=.673). Table 3 (Tab. 3) displays the items and factor loadings of the final scale. 

## Discussion of Study 1

The results of Study 1 show adequate psychometric properties of TAS. Three items had to be eliminated from the original item set. The remaining fourteen items can be distributed into three clear factors, theoretically derived largely from Rousseau [10]: TC, CIE and TAB. Of note, *Cooperation* could not be separated from* Information Exchange*, a finding that advances theoretic considerations: In short, simulated scenarios cooperation is - to a large extent - information exchange and, thus, one should not try to forceably differentiate them. 

Methodologically, this study provides evidence to reliably measure teamwork in the context of simulated ward-round scenarios of undergraduate medical students. This context had not been covered in the existing teamwork measurement instruments reviewed above. As past studies have shown [25], the transfer and application of general teamwork models to the medical field is not trivial. In the present study, the theoretical conceptualisation of teamwork (consisting of TC, CIE, and TAB) could be successfully demonstrated and evaluated in a simulated medical training setting. 

## Evaluation Study 2: Construct validation of TAS

The purpose of Study 2 was to assess the construct validity of TAS via comparison to an external criteria (i.e. clinical performance). Despite the variability of ward-rounds [19], each ward members’ role, objectives, and activities - and the typical sequence of activities - are predefined and can be called a collaboration script [23]. In our university clinic, a standard procedure for ward-rounds has been developed and implemented over the past years. Considerable time and effort has been spent by various experts to ensure that the best procedure became standardized and implemented in the clinic. This script (i.e. the structural requirements of the ward-round procedure) can be used both as an external index for the clinical performance of the team and to ensure proximity of TAS to actual teamwork. 

### Procedure

One hundred medical students (mean age=23.1, f=62%, m=38%) took part in the study for course credit. Twenty-five teams, each consisting of four members, performed in one of three ward-round scenarios. 

All scenarios were videotaped and rated in real-time with TAS by four independent raters. Two physicians and two undergraduate medical students volunteered for the study. This was done to test whether it is possible to train students in the use of TAS. All raters were trained in the use of TAS. The rating of one item was reversed as low ratings indicated good teamwork behaviour (“In situations where a team member needed help, it was not given to them.”).

In this study, the structural fulfilment of the requirements of the ward-round procedure is used as an index for clinical performance. In detail, it was analysed using a coding scheme for ward-round scripts focusing on predefined roles, objectives and activities of each member as well as the typical sequence of activities in a ward-round scenario, which had been taught to the students beforehand. In our university hospital, the *activities* for a for one patient normally consists of four parts. We used these four parts as a coding scheme with each part considered as fulfilled or not fulfilled. An example for a coding scheme for scenario one is presented in Attachment 1. In part one, the resident gives the relevant medical information to the senior physician. In part two, the nurse complements the doctor´s report. In part three, the patient is asked questions and examined by the resident. In part four, the resident and the senior physician discuss the treatment plan with each other and then with the patient. The overall *sequence of activities* consisted of the parts described in the order above. The *roles* were the professions active in the parts. *Objectives* for each part in each scenario for each of the professions were formulated (i.e. in one scenario the resident has the objective to state the T-negativations of the patient). For each scenario, one coding scheme was completed for every video by two members of the research team and received an interrater correlation of r= .99.

#### Statistical Analysis

The rating of all items was z-standardized. The scale’s internal consistency was checked using Cronbach’s α. Correlations of the scale were checked using Pearson correlation. 

## Results of Study 2

The descriptive data for study two is presented in Table 4 (Tab. 4). Cronbach’s a for the TAS was good (α=.78) for the physicians and moderate for the students (α=.69). The inter-rater correlation for the physicians was r_Physician1,Physician2_=.90 and for students r_Student1, Student2_=.36. For the physicians, significant correlations were obtained between all three factors: TC, CIE and TAB and clinical performance (CP) (r_TC,CP_=.60, r_CIE,CP_=59., r_TAB,CP_=.52) as well as between the total score and clinical performance (r_TAS,CP_=.64). 

However, for student raters, no significant correlation for the three factors with clinical performance (r_TC,CP_=.18, r_CIE,CP_=.04, r_TAB,CP_=.00.), nor between the total score and clinical performance, could be found (r_TAS,CP_=.04). This shows that TAS scores of physicians are more closely related to an external criterion. The correlations between clinical performance and TAS are summarized in Table 5 (Tab. 5).

## Discussion of Study 2

In the second study, TAS shows a significant relationship between TAS and clinical performance in ward-round scenarios (r_TAS,CP_=.64) for experienced physicians. Furthermore, TAS shows higher consistency in scoring by physicians than by medical students. Although Cronbach’s α show adequate values for student ratings, there seems to be no connection of ratings to the actual clinical performance of the team. Students´ scores seem to be consistent– but consistently wrong. Overall, the construct validity of TAS can be considered as adequate to high [26] for the use of physician raters in simulated ward-round scenarios of medical students.

Further validation is necessary to confirm our findings. Clinical performance analysed as scripted best-practice information by roles, objectives, activities, and sequence of activities is considered to be a promising direction for future validation steps. 

## General Discussion

The goal of this study was to develop an instrument for which the components and behavioral definitions derive from current teamwork research. Study 1 showed that the psychometric quality of TAS with trained raters was sufficiently high to measure teamwork. Both validation studies indicate good internal consistency for trained raters with teamwork experience. TAS has not yet been used, however, to validate teamwork trainings. Furthermore, there is an indication of construct validity for TAS: The comparison between physician and student raters indicated that the physicians’ ratings are more reliable than those of students, and only the ratings of the physicians had a significant relation to clinical performance as an external criteria. 

TAS differs in several key features from analogous instruments available in the current literature. First, TAS is an instrument specifically designed for the purposes of simulation-based training for medical students. Second, building on prior developments [12], [13], [14], [15], [16], [17], [21], TAS goes beyond the existing approaches to measure teamwork behaviour by defining each teamwork behaviour within a hierarchical, theoretical framework [10]. This structural approach will allow for behavioural feedback deriving specifically from the instruments’ subscales of TC, TAB and CIE, a consideration that has not been included in other instruments [12], [13], [14], [15], [16], [17], [18], [21]. Third, TAS also goes beyond the existing approaches by verifying construct validity in the early development stage. Through this theory-based development and preliminary construct validation, TAS seems closely related to actual teamwork of students. Although the findings of our studies are both in the context of simulated ward-rounds, the items within TAS are formulated in a generalized way to enable the measurement of simulated student teamwork beyond this medical context. 

## Limitations

The presented studies have two limitations: First, the instrument has been designed for undergraduate courses, which may limit the use of the instrument beyond the classroom. Second, despite careful selection of experts and independent ratings, study two was conducted with only four raters, which limits the findings to this small sample. This limitation is ameliorated by the stable internal consistency found for TAS in both studies. In addition, power analysis with a medium effect size (ρ=.30) indicates a sufficient measurement power for both study one (1- β=.82) and study two (1- β=.96) for the assessment points.

## Application

TAS, as presented here, is an easy-to-use instrument to evaluate behavioural components in simulated student ward-rounds via ratings of an experienced physician. To use TAS, only a short training video-based session is necessary to help anchor the ratings and TAS can then be used to guide further development of simulated teamwork scenarios. The final version of TAS is provided in Attachment 2. 

## Outlook

The validation showed that the behavioural components of teamwork, as drawn from the theoretical teamwork model by Rousseau [10], can provide a framework for measuring teamwork. As the focus of TAS lies on the applicability within short, simulated scenarios, the development of the scale focused on behavioural components of *Team Adjustment Behaviours* and *Task-related Collaborative Behaviours*. Certainly, actual teamwork is much broader (as the framework shows as well) and scales focusing on the other components could complement TAS (i.e. for the learning of long-term teams in an actual hospital setting). The factorial analysis was not able to separate items regarding Information Exchange and Cooperation but could separate Coordination. This can be explained by the high coordinative effort needed to operate in simulated scenarios. To date, coordinative behaviour was proven to serve as a protective barrier for medical errors in emergency medicine [18] but has not yet been of special interest in other hospital settings. As the possibility of measurement exists, future studies could question whether the same results regarding coordination can be found in other simulated hospital settings as well.

Future research should focus on questions regarding the use of the instrument as a source for feedback and subsequent learning achievement on an individual- and team-level. Such research could clarify whether TAS is sensitive to change. Since the current research has shown that training of students is not enough to adequately measure teamwork with TAS, future studies should address the question of whether a deeper understanding of teamwork in medicine and improved rater training can overcome this application barrier for novices. An analysis of the relation to the existing specific scales for emergency medicine [15], [16], surgery [12], or anaesthetics [13] has not yet been done but could help to further clarify the range of application for TAS. Methodologically, the construct validation of behavioural observation of TAS with clinical performance analysed as detailed script information by *roles, objectives, activities*, and *sequence of activities* is considered to be a promising direction for future research.

With further validation, TAS can guide the development and implementation of simulation-based teamwork training programs in medical education [8], [9]. We hope that TAS can contribute to the further improvement of simulation-based teamwork training and, ultimately, to safer healthcare.

## Acknowledgement

*The initial ideas for items 1, 2, 6, 7, 8, 9, 13, 14, 15 derived from the instrument by Weller et al. [16], the ideas for items 3, 4, 12, 17, 16 derived from Malec et al. [17], the ideas for item 10, 11 and 15 derived from Fletcher et al. [13] (see Table 2 (Tab. 2)).

The first author is thankful for a DAAD grant during the time of the manuscript preparation. The application of the scale is free of charge but the authors appreciate a short note when doing so.

## Competing interests

The authors declare that they have no competing interrests.

## Supplementary Material

Attachment 1: Example of the coding scheme used for scenario one

Attachment 2: Teamwork Assessment Scale (TAS) with a 5 point Likert-scale

## Figures and Tables

**Table 1:**
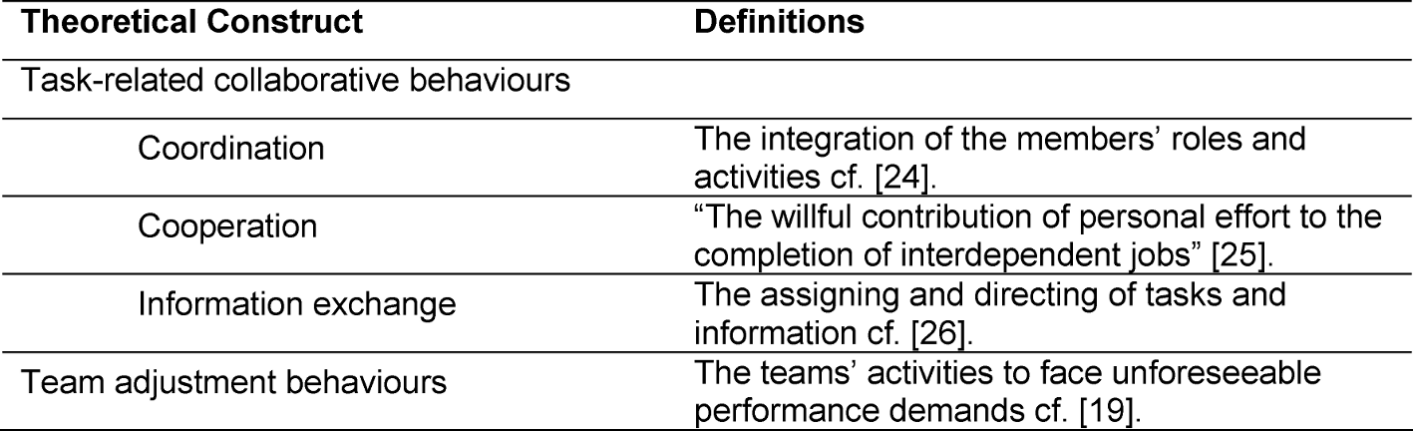
Theoretical foundation and definitions for the development of the measurement instrument

**Table 2:**
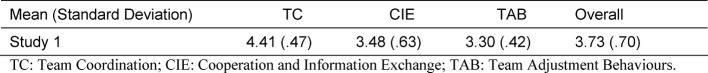
Descriptive data of study 1 (Overall range 2-5).

**Table 3:**
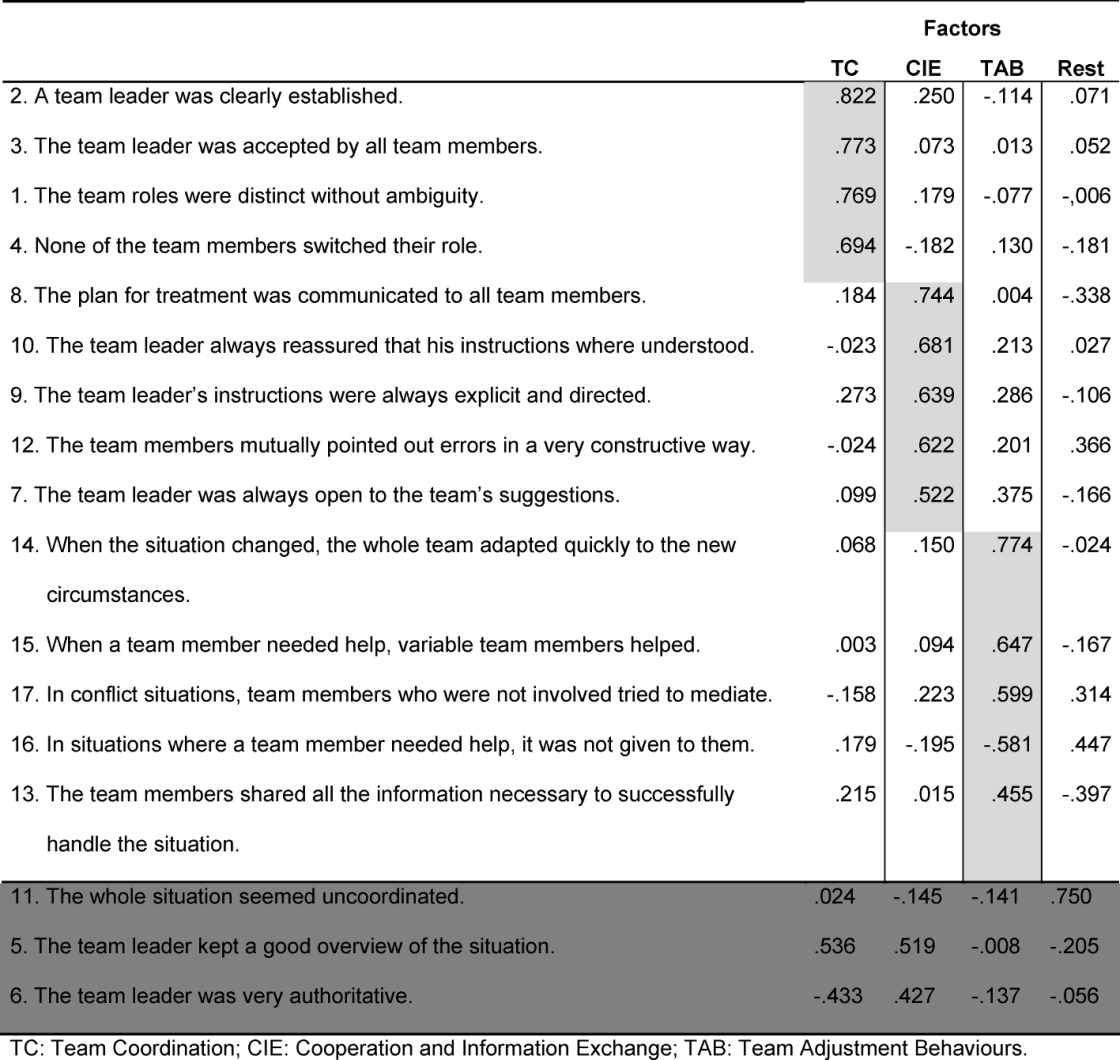
Result of the factor analysis in study 1.

**Table 4:**
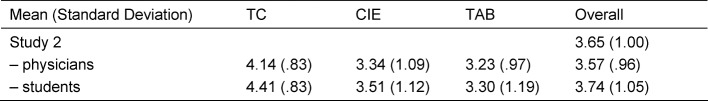
Descriptive data of study 2 (Overall range 1-5).

**Table 5:**
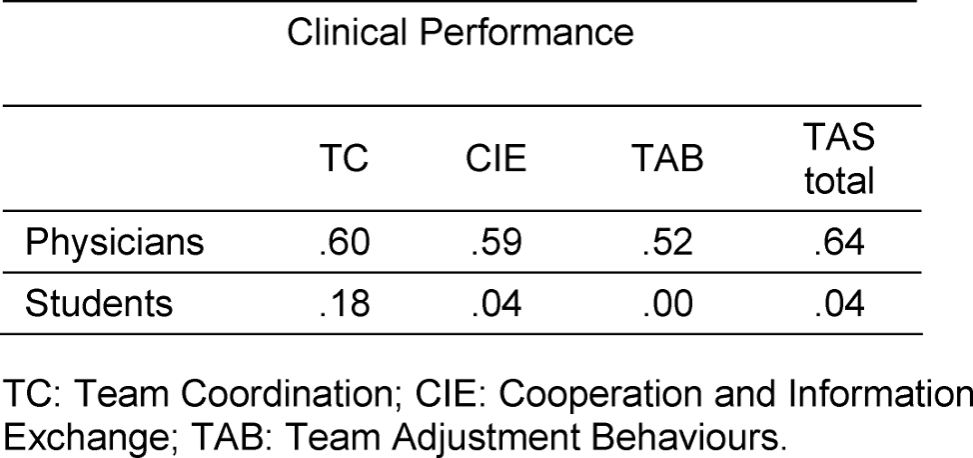
Summary of factors correlations between clinical performance.
