# Supplementary material for: The Teamwork Assessment Scale: A Novel Instrument to Assess Quality of Undergraduate Medical Students' Teamwork Using the Example of Simulation-based Ward-Rounds
Source: GMS Z Med Ausbild. 2015 May 13;32(2):Doc19. doi: 10.3205/zma000961 (PMC4446650; doi:10.3205/zma000961)
Supplement: Attachment 1: Example of the coding scheme used for scenario one [file ZMA-32-19-s-001.pdf]

Attachment 1: Example of the coding scheme used for scenario one

Part 1: Resident gives relevant medical information to senior physician	Yes	No
Part 2: Nurse complements the doctor´s report	Yes	No
Part 3: Patient is asked questions (and examined) by the resident.	Yes	No
Part 4: Resident and senior physician discuss the treatment plan with each other and then with the patient.	Yes	No
Sequence of activities: Part 1-4 were carried out in correct order	Yes	No
Roles: Every person acted concurrent to their role<br>nurse as nurse, resident as resident, senior physician as senior physician	Yes	No
Objectives: The objective of the scenario was fulfilled<br>Scenario 1: Resident states T-negativations of the patient	Yes	No
