# Supplementary material for: The Teamwork Assessment Scale: A Novel Instrument to Assess Quality of Undergraduate Medical Students' Teamwork Using the Example of Simulation-based Ward-Rounds
Source: GMS Z Med Ausbild. 2015 May 13;32(2):Doc19. doi: 10.3205/zma000961 (PMC4446650; doi:10.3205/zma000961)
Supplement: Attachment 2: Teamwork Assessment Scale (TAS) with a 5 point Likert-scale [file ZMA-32-19-s-002.pdf]

Attachment 2: Teamwork Assessment Scale (TAS) with a 5 point Likert-scale

TEAMWORK ASSESSMENT SCALE

J. KIESEWETTER*+, M.R. FISCHER*

*INSTITUT FÜR DIDAKTIK UND AUSBILDUNGSFORSCHUNG IN DER MEDIZIN, KLINIKUM DER LMU MÜNCHEN

+MUNICH CENTER OF THE LEARNING SCIENCES, LMU MÜNCHEN

	Completely Disagree				Completely Agree
The team roles were distinct without ambiguity.	①	②	③	④	⑤
A team leader was clearly established.	①	②	③	④	⑤
The team leader was accepted by all team members.	①	②	③	④	⑤
None of the team members switched their role.	①	②	③	④	⑤
The team leader was always open to the team's suggestions.	①	②	③	④	⑤
The plan for treatment was communicated to all team members.	①	②	③	④	⑤
The team leader's instructions were always explicit and directed.	①	②	③	④	⑤
The team leader always reassured that his instructions where understood.	①	②	③	④	⑤
The team members mutually pointed out errors in a very constructive way.	①	②	③	④	⑤
The team members shared all the information necessary to successfully handle the situation.	①	②	③	④	⑤
When the situation changed, the whole team adapted quickly to the new circumstances.	①	②	③	④	⑤
When a team member needed help, variable team members helped.	①	②	③	④	⑤
In situations where a team member needed help, it was not given to them.	①	②	③	④	⑤
In conflict situations, team members who were not involved tried to mediate.	①	②	③	④	⑤
